# Measuring Early Hearing Detection and Intervention (EHDI) Quality across the Continuum of Care

**DOI:** 10.5334/egems.239

**Published:** 2018-07-24

**Authors:** Xidong Deng, Terese Finitzo, Subhash Aryal

**Affiliations:** 1 Centers for Disease Control and Prevention, US; 2 OZ Systems, US; 3 UNT Health Science Center, US

**Keywords:** Clinical Quality Measure, Electronic Health Records, Public Health Surveillance, hearing, reporting, data collection

## Abstract

Improving quality measurement while reducing costs helps public health programs identify and better support critical aspects of the care and services delivered to the patients they serve. This is true for state-based early hearing detection and intervention (EHDI) programs as they strive to develop robust clinical quality measures to help track the quality of hearing health services provided during the EHDI processes. Leveraging today’s electronic health records and public health surveillance system functionalities, state reporting requirements facilitate and yield efficient collection and analysis of data for quality measurement. In this study, we tested three EHDI quality measures endorsed by the National Quality Forum using a retrospective sample of more than 1,100,000 newborns from 3 states using electronic health data available in the state EHDI Information Systems (EHDI-IS). The results of the analysis reported herein from a large multi-state cohort provide a “real life” benchmark for future quality improvement projects and of where EHDI stands today. Reflecting on these findings, suggestions are posed for enhancing the EHDI quality measures in future updates.

## Introduction

Congenital hearing loss affects two to three infants per 1,000 live births and, if undetected, can delay speech, language, and cognitive development [1]. The goal of state-based early hearing detection and intervention (EHDI) programs is to facilitate early identification and maximize language and literacy for children who are deaf or hard of hearing (D/HH). The newborn nursery provides a near universal, captive population to achieve this goal. Hospital point of care screening has been in place for nearly two decades. Yet the success of EHDI programs ultimately depends on the availability, quality, and equity of care and services provided not just during the nursery-based screening but at sequential points of screening and follow-up, including diagnostic evaluations and enrollment into early intervention. The benefits of early hearing detection accrue when these subsequent steps are timely and efficient. Recent national surveys show gaps in the process and indicate that additional improvements may be helpful [2].

As stated in a recent article on the importance of the pediatric quality measurement program in advancing children’s health care [3], “Without consistent measurement and reporting, hospitals and other providers will be subject to a cacophony of measures from other insurers, and parents will be left without comparable information to engage in their child’s care.” As EHDI programs advance, robust measures may be beneficial to hospitals and providers as they assess the quality and value of the care they provide. Several Clinical Quality Measures (CQMs) were developed by the Centers for Disease Control and Prevention (CDC) and endorsed by the National Quality Forum (NQF) [4] to help track the quality of hearing health care services provided in the EHDI processes. These include EHDI-1a (NQF #1354): Hearing Screening Prior to Hospital Discharge; EHDI-3 (NQF #1360): Audiology Evaluation prior to 3 months of age; and EHDI-4 (NQF #1361): Signed Individual Family Service Plan (IFSP) prior to 6 months of age. These measures have been used in public health reporting initiatives, provider incentives, and accreditation and certification programs to increase provider accountability and promote informed consumer choice. They have a long history in EHDI quality, appearing as important quality indicators (QIs) in the Joint Committee on Infant Hearing (JCIH) 2007 Position Statement [5]. In particular, all three measures are reported to CDC by state EHDI programs in the annual Hearing Screening and Follow-Up Survey (HSFS) [6]. The EHDI-1a measure was chosen by the Centers for Medicare and Medicaid Services (CMS) as one of the Meaningful-Use Stage 2 quality measures for eligible hospitals in the Clinical Process/Effectiveness National Quality Strategy Domain [7]. It has also been adopted by the Joint Commission as one of the 13 electronic CQMs for hospital accreditation [8]. EHDI-3 has been included in the CMS Core Set of Children’s Health Care Quality Measures since 2016 [9].

Despite adoption of the EHDI CQMs, there is a lack of a consistent data collection and only limited information about measure use and results are available to CDC and other stakeholders. Adopting agencies currently use the measures in different ways and the measure is reported only at specific granularity or for a specific sample population. For example, the Child Core Set uses the EHDI-3 measure only for children enrolled in Medicaid and the Children’s Health Insurance Program (CHIP). In the case of the HSFS, the measures are reported at the aggregated state level only. In addition, although the three EHDI CQMs address different stages of the EHDI processes, they are closely related to each other and ideally need to be assessed at the same time for the same sample population to provide a cohesive and comprehensive estimate of the overall quality of EHDI services across the entire continuum of care.

A consistent data collection mechanism with centralized analytic capability could help address these gaps in knowledge. With the evolution of today’s electronic health records (EHRs) and public health surveillance system functionality, state reporting requirements facilitate and yield efficient collection of data and reduce data collection barriers and burdens for hospitals and providers to report quality measure information. State-based EHDI surveillance and tracking information systems, or EHDI-IS, are confidential, computerized, population-based systems that collect and consolidate newborn hearing screening, follow-up care, and service data from clinical and early intervention service providers. EHDI-IS support effective tracking and surveillance by ensuring D/HH infants receive the services that can improve their outcomes and include the data that can be used to meet various quality measurement and reporting requirements.

In this project, we evaluated the three NQF endorsed CQMs across the EHDI continuum of care using a retrospective sample of more than 1,000,000 newborns from three EHDI state programs (denoted as state A, B, and C in this paper). The records were obtained from the participating states’ EHDI-IS. As part of this review, we calculated the three measures and compared outcomes for the cohorts from the three states. We also examined each measure definition and reviewed data elements, the inclusion/exclusion criteria, and the code sets used. The results of the analysis of the data reported from this multi-state cohort provide a “real life” benchmark for future QI projects and provide a first benchmark of where EHDI stands today. We also compared measure outcomes among different population groups (e.g., by birth location or hospital size). Based on these findings, we have made suggestions for enhancing the EHDI CQMs in future updates that can further improve specific stakeholders’ performance.

## Methods

The patient cohort for this study is comprised of infants born from 1/1/2013–6/30/2015 as documented in three states’ EHDI-IS. A total of 1,124,377 de-identified patient records were extracted. Each state utilizes the same core tracking and information management system: eSP™ provided by OZ Systems. Each state has customized the system to meet specific state requirements. Each EHDI-IS was employed to contribute data for the three NQF measures.

Patient-level newborn hearing screening and follow-up data were extracted from the states’ EHDI-IS and exported into Microsoft Excel.® The privacy of the patients’ records used for the analysis was maintained by removing the name identifiers from each record. An artificial identifier, in the form of a unique number assigned to each record by the OZ eSP™ EHDI-IS, was used as the key identifier for each record. Project staff reviewed the raw data to ensure records were de-duplicated and coded consistently across the three participated states.

The Statistical Analysis System (SAS 9.4, Cary, NC) tool was chosen for analysis and evaluation for each measure definition. To maintain experiment-wise type I error rates, our primary statistical test involved omnibus testing using the chi-square test for overall differences between the three states. Univariate z-tests were then conducted to further compare the proportion between the three states. To maintain the experiment-wise type I error rate (α) at 5 percent, the Bonferroni adjustment was used. As a result, only *p*-values < 0.016 (α/3) were considered significant. To examine the differences in the number of infants screened before discharge based on hospital birth census, hospitals with annual birth rates above and below the median number of births for the facilities in the data set were categorized. A comparison was made of the proportion of newborns screened in facilities with more than the median number of births versus below the median number of births. Birth location was classified as urban and rural based upon the birth facility’s ZIP code, and classification was provided by the SAS applying Metropolitan Statistical Areas.

## Results

NQF #1354: Hearing Screening Prior to Hospital Discharge

 

This measure assesses the proportion of births that have been screened for hearing loss before hospital discharge.

Table 1 shows the definition of the EHDI-1a measure at the time of the study, during which time several updates to the measure were under consideration. One update under consideration was excluding infants who did not receive hearing screening due to parent refusal from the denominator population. The decision by families to refuse hospital newborn hearing screening, while infrequent, is recognized as a valid reason for the infant not receiving service, and supports the 2007 JCIH position statement benchmark specifying that “the EHDI system should be family-centered with infant and family rights and privacy guaranteed through informed choice, shared decision-making, and parental consent in accordance with state and federal guidelines [5].” Circumstances of parent refusal of screening is typically documented in the EHDI-IS that was used in this project, so that the element can be extracted for this measure. Further, applying delineation of parental refusal change to this measure prevents the inaccurate reporting of infants as not screened (i.e., inaccurate attribution of failure to conduct screening). Therefore, in this project we added parent refusal to the denominator exclusion criteria.

**Table 1:** NQF #1354 (EHDI-1a): Hearing Screening Prior to Hospital Discharge.


**Measure Description**	NQF #1354 assesses the proportion of births that have been screened for hearing loss before hospital discharge.
**Measure Definition**	
Denominator	All live births discharged during the measurement time period born at a facility.
Denominator Exclusions	Patient deceased prior to discharge and has not received hearing screening.
Numerator	All live births during the measurement time period born at a facility and screened for hearing loss prior to discharge, or not being screened due to medical reasons or medical exclusions.


### State Outcomes

A combined total of 1,124,377 infants were assessed for this measure. As seen in Table 2, the infants were categorized into three groups: initial population (denominator), screened, and excluded because of early death or parent refusal. Thus 9,323 newborns of the original cohort of 1,124,377 were excluded: 4,568 (48.9 percent) due to parental refusal and 4,755 (51.0) because of reported early death. A comparison of the states’ data found that over 98.9 percent of infants in each state had hearing screening conducted before discharge from the birthing facility. A statistically significant difference was observed between states (p < 0.0001).

**Table 2:** NQF 1354 – Hearing Screening Prior to Hospital Discharge.

State	Initial Population (Denominator)	Excluded	Total Eligible	Numerator	Measure Score (%)

**A**	169,299	1,061	168,238	166,431	98.9
**B**	31,063	177	30,886	30,597	99.0
**C**	924,015	8085	915,930	911,569	99.5
**Total**	1,124,377	9323	1,115,054	1,108,597	99.4

### Effects of Hospital Size and Birth Rate Differences in Hearing Screening Rates

The median number of births by hospital was 1,427 in the cohort over the course of the study period. When differences in the number of infants screened for hearing before discharge in hospitals was assessed, the screening rate in hospitals that had more than the median number of births was 99.6 percent. Facilities with fewer than the median number of births screened 96.0 percent of newborns. The difference was statistically significant (p < 0.0001).

When hospitals with fewer than 100 births were excluded from analysis, the screening rate in facilities with fewer than the median number of births improved slightly to 96.3 percent, though the difference remained statistically significant (p < 0.0001).

### Impact of Birth Location (Urban versus Rural) on Screening Rates

We used the 2010 Census definition of Urbanized Areas [10] to breakdown the birth cohort into urban (50,000 or more people in a geographic area) and rural defined as (<50,000 people in a geographic area). Using this definition, 93.1 percent (1,035,470/1,111,779) of the birth cohort were born in urban facilities and 6.9 percent (76,309/1,111,779) rural.

Overall, 99.6 percent of newborn babies in urban hospitals and 98.8 percent of newborns in rural hospitals received hearing screening. There was a statistically significant difference (p < 0.0001) found in hearing screening between urban and rural areas. A comparison of screening by state showed a statistically significant difference was observed for all three states (p < 0.0001).

 

NQF #1360: Audiological Evaluation No Later than 3 Months of Age

 

Table 3 shows the measure definition for EHDI-3. There is an inherent discrepancy in the measure definition: it was stated in the measure description that the measure assesses audiological evaluation no later than 3 months of age. However, the description of the numerator section implies that not just an evaluation, but a confirmatory diagnosis must be made for the child to be included. Not every diagnostic evaluation results in a diagnosis on the same day, so varying interpretation of the measure definition will yield different results.

**Table 3:** NQF #1360 (EHDI-3): Audiological Evaluation by Three Months.


**Measure Description**	NQF #1360 assesses the percentage of newborns who did not pass hearing screening and have an audiological evaluation no later than 3 months of age.
**Measure Definition**	
Denominator	Denominator contains the number of infants born during the time window who have not passed (“Fail/Refer”) hearing screening.
Denominator Exclusions	Patient deceased: Patient has expired prior to 91 days of age.
Numerator	Numerator contains the number of infants born during the time window who have not passed (“Fail/Refer”) hearing screening and whose age is less than 91 days at audiological diagnosis.


These three EHDI-IS include both date of evaluation and diagnosis, so we were able to calculate the measure score using either interpretation, while using the number of infants that did not pass the final screen (n = 14,528) as the denominator. Some states employ a multi-staged screening protocol: if a child failed the initial hospital screening, it is required that an outpatient follow-up screening be performed before referring the child to diagnostic evaluation. Results of the follow-up screening are also reported to the EHDI-IS and we use them in combination of the initial hospital screening results to determine the “final” screening result. Of those infants who failed the final screening and thus needed an audiologic evaluation to rule out permanent hearing loss (n = 14,528), 2,374 (16.3 percent) received diagnostic evaluation, out of which 86.8 percent (n = 2,060) were documented as having completed the evaluation with a confirmatory diagnosis of permanent hearing or normal hearing. There were 1,535 infants who received diagnostic evaluation by 91 days of age, and 1,355 obtained a confirmatory diagnosis by 91 days of age, which yielded the measure scores, 10.6 percent and 9.3 percent, under the two different interpretations, respectively. Table 4 shows the number breakdown for each of the three participating states.

**Table 4:** Audiological Evaluation and Diagnosis by State.

State	Failed Final Hearing Screening	Received an Audiological Evaluation	Received an Audiological Evaluation by 91 Days	Received an Audiological Diagnosis	Received an Audiological Diagnosis by 91 Days

**A**	2,098	369	196 (9.3%)	323	171 (8.2%)
**B**	897	62	37 (4.1%)	54	34 (3.8%)
**C**	11,533	1,943	1,302 (11.3)	1,683	1,150 (10.0%)
**Total**	14,528	2,374	1,535 (10.6%)	2,060	1,355 (9.3%)

### Age of Audiological Diagnosis

Regardless of age, a total of 14.2 percent (2,060/14,528) of infants in the measure denominator population had documentation of o receipt of a diagnosis. Age of diagnosis was found to be within 91 days for 65.8 percent (1,355/2,060). An additional 21.4 percent (441/2,060) received a diagnosis between 91 and 180 days. Another 9.1 percent (187/2,060) received a diagnosis between 181 and 365 days and the remaining 3.7 percent (77/2,060) received a diagnosis after more than 365 days. At the time of analysis, these numbers represent all data received from audiologists and entered into the states’ EHDI-IS.

 

NQF #1361: Signed Part C Individual Family Service Plan (IFSP) before 6 Months of Age

 

As shown in Table 5, to determine if an infant who is D/HH is not just referred but also connected to early intervention services, the NQF measure 1361 stipulates that there must be a record of a parent’s signed IFSP as required by federal and state IDEA Part C laws. Of the total of 772 infants identified at any age with permanent hearing loss in this cohort, only 26.7 percent (206/772) had a signed IFSP, of which 114 had an IFSP signed within 6 months, yielding a measure score of 14.8 percent (114/772 across all three jurisdictions). Similarly, 7.3 percent (56/772) had an IFSP signed between 6 months and 1 year, 4.7 percent (36/772) signed after 1 year, and the remaining were considered loss to follow-up or loss to documentation. There is no statistically significant difference among the three states in the percentage of infants having a signed Part C IFSP by six months (p = 0.1333).

**Table 5:** NQF #1361 (EHDI-4): Signed Part C Individual Family Service Plan (IFSP) by 6 Months of Age.


**Measure Description**	NQF #1361 assesses the proportion of infants with permanent hearing loss referred and receiving an Individual Family Service Plan (IFSP) to receive intervention services under Part C of the Individuals with Disabilities Education Act (IDEA) that is signed by the time the infant is 6 months of age.
**Measure Definition**	
Denominator	Denominator contains number of infants born during the time window diagnosed with permanent hearing loss.
Denominator Exclusions	Patient deceased: Patient has expired prior to 181 days of age.
Numerator	Numerator contains the number of infants born during the time window who have been diagnosed with permanent hearing loss, whose age is less than 6 months at the time of having a signed Individual Family Service Plan (IFSP) to receive intervention services under Part C of the Individuals with Disabilities Education Act (IDEA).


### Impact of Race and Ethnicity

Race and ethnicity have long been considered as important factors when assessing disparity in the receipt of health care services, including audiological diagnostic care. Only 0.7 percent (8,133/1,124,377) of the infants in the entire cohort had ethnicity information documented in the EHDI-IS. Race information was available for 36.4 percent (409,662/1,124,377) of the cohort, yet the information was not deemed to be reliable, as most of the documented values were “Other,” which did not offer insight into specific values that align with the census reporting for race. The state programs contributing to this project did not utilize an electronic import of this information from an electronic health record for the reporting years. Moreover, with the extent of information considered essential for appropriate early intervention care, race and ethnicity, though often required, were not critical elements considered; thus, the race and ethnicity data quality and quantity are poor in this cohort and not reliable. For these reasons, a decision was made not to perform a statistical analysis on any of the measures based on race and ethnicity.

## Discussion

The concept of EHDI quality benchmarks was first introduced by JCIH in its year 2000 position statement [11]. Benchmarks are meant to change with continuous quality improvement. Because EHDI programs were relatively new at that time, the year 2000 position statement included benchmarks based on existing data and suggested others in areas for which published data were not available. The intention was to identify key indicators of EHDI quality and measure a result in relation to a stated benchmark. QIs would be monitored using well-established practices of statistical process control to determine program consistency and stability [12]. If the QIs reveal that a program is not meeting the benchmark, sources of variability could be identified and corrected to improve the process [13].

 

NQF #1354: Hearing Screening Prior to Hospital Discharge

 

The analysis of screening results from this cohort prior to discharge showed that the data are robust and consistent within states. More than 99 percent of reported newborns are screened, exceeding the initial JCIH benchmark of 95 percent. Thus, a new benchmark could be established to encourage continued improvements. Results also suggested that there are challenges in completing screening in smaller facilities and in rural areas, and thus help identify areas of program improvement. There are, however, sources of variability and issues related to the accuracy of the measure number. First, the entire hospital birth cohort may not be accounted for in the denominator since it is possible that babies who are not screened may not be entered into the EHDI-IS if manual entry is required. Standardizing how the hospital births are reported so that there is a consistent, accurate, and complete representation of births may strengthen the measure definition. This can be achieved through implementation of electronic demographic reporting from the hospital’s electronic medical record (EMR) to the state’s EHDI-IS. Many states do not require auditable data on demographic data (such as race and ethnicity) or hearing screening for each birth in each hospital. Without verifiable data from the EMR, state agencies responsibility for health cannot know that babies not screened are entered into the EHDI-IS. CDC recognized this limitation and, in late 2011, worked with the standards organization, *Integrating the Healthcare Enterprise*, to complete a technical profile called Newborn Admission Notification Information (NANI) [14]. The purpose of NANI is to assure that a birthing facility’s EMR information is available to public health in real time to alert if a baby’s hearing screening (or other screening) is complete. NANI focuses on data elements important to newborn identification (e.g., time of birth, multiple births). In 2015, some but not all birthing facilities in each of the three states were using NANI. In those facilities using the NANI protocol, the baby’s hearing health care audit trail accurately reflects the birth and the screening status. As of January 2017, NANI has been implemented in an average of 40 percent of the hospitals in eight states, as well as the three states in this analysis. For a state cohort with NANI in place, aligning the denominator more closely to the birth registration number has proven to be useful.

Refining the NQF #1354 measure definition with regard to adding parental refusal to the denominator exclusion criteria could also be beneficial. Exclusion due to parental refusal accounted for 0.49 percent of the 0.83 percent of the initial population that was excluded from the denominator. Knowing the percent of families in the cohort that refused screening is valuable information. If the percentage were to increase in a hospital system, the state agency might wish to discuss protocols with hospitals. The medical home may have access to this information in order to advise parents. Also, as mentioned above, this is in keeping with the JCIH 2007 recommendation for family-centered care. When death and refusal numbers are excluded from NQF #1354, more than 99 percent of the cohort was screened in each state. If reliability is defined as stable, replicable, and consistent results, the NQF measure #1354 in this study design yields reliable results.

 

NQF #1360: Audiologic Evaluation by 91 days of age

 

NQF #1360 is the percentage of newborns that did not pass hearing screening and had an audiological evaluation/diagnosis no later than 3 months of age. Outside the birthing facility where hospitals are successfully screening newborns before discharge, information for this measure is obtained from provider offices, raising new issues. The JCIH 2000 Position Statement benchmark for follow-up care was not based on actual information as there was concern that few high-quality data were available prior to its publication. In 2000, JCIH set the benchmark to 95 percent of babies needing follow-up care receiving it by three months of age. Few hospitals and no states achieved this benchmark.

There are limited verifiable and auditable data at the state level regarding this measure, so the evidence is not of high enough quality. Specifically, verifiable data mean

- each eligible newborn is included in the denominator;
- the number and names of patients being referred (do not pass the hearing screen) are known; and
- each infant can be tracked to a care conclusion, either confirmation of hearing loss or the confirmation of normal hearing.

More than 14,000 infants did not pass hearing screening and needed follow-up audiologic evaluation. According to the data from this cohort, 9.3 percent of these infants received an audiologic diagnosis by 3 months of age during the time period of this evaluation. Additional infants may have been tracked to an outcome by state staff separately after data were collected for this evaluation. The remainder would fall in the “loss to documentation” or “loss to follow-up” categories. In part this is due to the delay in reporting this measure by audiologists, resulting in underreporting, even when the family is referred appropriately and connected to care. More infants likely received care, but the state EHDI program did not learn about it and it was therefore not documented.

Audiologists often do not report results consistently, even when there is legislation in place to require it. Reporting is a financial burden and providers note that too much information is requested. Reviewing the required data elements and reducing what is needed by EHDI programs to a minimum is one strategy some states are trying. This would ease the provider’s reporting burden. Additional strategies that could be explored include simplifying the task via electronic reporting, providing payment for reporting, and leveraging insurance claims or Medicaid claims databases. Nearly half of the cohort is Medicaid eligible and linking EHDI-IS with state Medicaid system could yield enhanced findings.

For this evaluation, we did not consider parental reports of outcome to be sufficiently comprehensive and so they were not included in the analysis, reducing the cohort. Only reporting by the audiologist, the professional considered to have the knowledge, skills, and equipment to provide standardized information for objective measures, contribute to the measure to provide the strongest data. We also attempted to maintain the concept of auditable data for quality purposes. If parental report is an allowable report on audiology outcome, it could be included in the measure definition as such.

Changing the measure description to “Audiological diagnosis by three months of age” may also address some reporting issues. Although including an infant whose evaluation was still in process could increase the numerator, it does not reflect the true need of the patient for receiving timely care and any subsequent service. Many pediatric audiologists prefer ongoing assessments and refinements to a single diagnosis and date. Understandably, providers want outcomes to be valid and reliable, often a challenge with children’s varying cooperation and medical conditions, so many infants remain in an unknown category despite two or three evaluations. Communicating to audiologists that reporting to public health agencies requires the best diagnostic information available at the time of reporting helps reduce the unknown outcomes. It is preferable to identify an infant with a permanent hearing loss than categorize that infant as “in process” or unknown.

Given the low performance on this measure, the measure definition could be modified further. Currently the measure decision was binary, recorded simply as yes or no. It does not address the range of ages for audiologic diagnoses and it does not include those diagnoses that occurred after 91 days, though we did report them above (see Results). This raises the question of whether this is the correct metric or at this stage of EHDI program development, or if we need more information on diagnostics on a continuum.

There are many variations in the measure definition and data collection/reporting processes that would affect the measure implementation. For example, whether “in-process” patients (those who have received an evaluation but no confirmatory diagnosis has been made) ought to be included in the numerator, and whether parental reports should be accepted for the purpose of quality measure reporting. Determining the sources of variability that have made this measure intractable over many years and allowing stakeholders to open a dialogue on data for timely audiologic decision-making and quality measurement could be beneficial.

 

NQF #1361: Signed Part C Individual Family Service Plan (IFSP) before 6 Months of Age

 

NQF #1361 stipulates having a signed IFSP, as required by federal and state IDEA Part C laws. An IFSP signed within six months is considered best practice for children’s developmental purposes. Of families in this cohort who received a hearing loss diagnosis, more than half received early intervention services under federal and state IDEA Part C.

Like NQF #1360, 1361 proved challenging to calculate, as the three states collected different data and used different data definitions. Of 772 infants with permanent hearing loss in this cohort, only 14.8 percent (114/772) had a signed IFSP within six months. First, different definitions among and between states confound data analyses. Only state A had a data element field in the EHDI-IS for signed IFSP. In state C, minimal data are available despite a direct connection from their EHDI-IS to the Part C Agency. The state C Part C program has determined that the Family Education Rights and Privacy Act (FERPA) rules prevent Part C programs from sharing which infants were enrolled in Part C. State B used date of referral to IDEA Part B to infer Part C enrollment. Additional standardization of NQF #1361 in how the states capture and define these data elements may be beneficial. Second, without accurate source data, the outcome data will not accurately reflect the measures as they are defined.

## Conclusion

Having robust quality measures is a step toward EHDI programs demonstrating a return on investment for the newborn, the family, and the health and education systems that design, finance, and implement these programs. The testing of the three EHDI CQMs using electronic health data available in state EHDI-IS indicated that the results for NQF #1354 are reliable and valid to the extent that similar methods are used to provide an accurate denominator. For NQF #1360 and 1361, our results are repeatable, but not valid in describing the “real world,” because of the variability in data capture that is dependent on states’ definitions. This will make applying both 1360 and 1361 challenging in other populations without further clarification of the definitions. This evaluation highlights the need to better identify the lost categories of patients. Innovative strategies, such as the use of standardized electronic data transmission across health systems, could possibly improve data collection. Leveraging electronic data and enabling cross-sectional system communication are especially important to EHDI to ensure timely interdisciplinary service delivery, which will help support families of children with or at risk of D/HH within the critical early childhood brain and social-emotional development paradigms.
